# A Fluoroscopy-Free Ablation Workflow for Persistent Atrial Fibrillation Using a Pentaspline Pulse Field Ablation Catheter Guided by Left-Sided Intracardiac Echo Imaging and Electroanatomic Mapping: A Case Series

**DOI:** 10.3390/jcdd12100412

**Published:** 2025-10-17

**Authors:** Adam Mohmand-Borkowski

**Affiliations:** Department of Cardiology, Cape Cod Hospital, 25 Main Street, Hyannis, MA 02601, USA; amohmand@capecodhealth.org

**Keywords:** pulse field ablation, intracardiac echocardiography, persistent atrial fibrillation, pentaspline catheter, fluoroscopy-free ablation

## Abstract

Background: Pulse field ablation (PFA) is a novel ablation technology with efficacy and safety, potentially making it a preferred ablation technology for persistent atrial fibrillation (AF). There is no optimal procedural workflow established to optimize efficacy, limiting the number of PFA applications and risks of the procedure. Due to the importance of optimal catheter–tissue contact for effective pulse field ablation, a workflow combining superior left atrial intracardiac echo (ICE) imaging and electroanatomic mapping (EAM) is an attractive strategy for PFA of persistent AF. Methods: A detailed procedural workflow was developed for fluoroscopy-free PFA using a pentaspline ablation catheter supported by left atrial ICE and EAM, and a case series of its execution in 30 consecutive patients with persistent AF is presented. All patients underwent pulmonary vein and posterior wall isolation as the index procedure, followed by additional ablation targeting non-pulmonary vein triggers and other inducible atrial arrhythmias. Results: Left atrial ICE imaging and EAM guided procedure resulted in successful isolation of the pulmonary veins and posterior wall in all patients, with additional ablation of spontaneous arrhythmias or non-pulmonary triggers if induced. There were no major complications of the procedure. Average procedure times and short-term efficacy were comparable with reported PFA outcomes using traditional imaging techniques. Conclusions: Fluoroscopy-free PFA guided by left-sided ICE for persistent AF can be performed with superior catheter–tissue contact imaging in a safe manner with a comparable procedural time and short-term efficacy as reported with the use of other imaging modalities.

## 1. Introduction

Catheter ablation has become the gold-standard therapy for rhythm control of AF with pulmonary vein isolation (PVI) being the cornerstone of ablation for paroxysmal AF. With progression of atrial fibrillation to persistent to long-standing persistent, the ablation procedure becomes less effective with controversies regarding the optimal ablation strategy. In a recent meta-analysis of different ablation strategies for persistent AF, which included 52 studies between 2004 and 2024 with more than 9000 patients, PVI with posterior wall box isolation and extra-pulmonary vein triggers ablation was found to be the most effective strategy [1].

Traditional energy sources using radiofrequency and cryoablation for persistent atrial fibrillation had been limited by the risk of collateral damage such as esophageal injury with left atrial posterior wall ablation (LAPW), with mitigation strategies frequently resulting in decreased durable lesion placement and procedural success rates. PFA, a novel nonthermal alternative ablation technology, uses electroporation and results in tissue-specific differences that allow ablation of atrial substrates with a limited risk of collateral damage, making it a very suitable technology for PVI and LAPW isolation.

With data published in large studies and registries supporting PFA efficacy and safety [2,3,4,5,6], the use of this technology has expanded for persistent atrial fibrillation treatment, with Farapulse (Boston Scientific, Marlborough, MA, USA) pentaspline PFA catheter being most frequently used. Zero- or minimal-fluoroscopy ablation techniques based on the use of ICE and 3-dimensional EAM had been previously incorporated into ablation workflows by some operators for radiofrequency and cryoablation to limit radiation exposure, and the safety of these approaches was confirmed in published prospective studies and meta-analyses [7,8].

Compared to ICE imaging from right atrium (RA), imaging from the left atrium (LA) produces superior images of LA structures and PV during the ablation procedure, and the safety of LA ICE as compared to RA ICE during RFA for AF has been previously reported [9]. Furthermore, findings from animal studies showed that guiding catheter–tissue contact by imaging rather than purely by the degree of contact force is crucial for the delivery of PFA [10]. Due to the critical importance of catheter–tissue contact for effective ablation using PFA and the limitations of fluoroscopy and EAM for this purpose, a workflow that combines intracardiac echo imaging and EAM appears to be an attractive strategy for persistent atrial fibrillation ablation using PFA. However, limited reports on ICE-guided PFA of atrial fibrillation with a fluoroscopy-free approach have reported the use of ICE imaging from the right atrium [11,12], while others leave the ICE imaging startegy to the operator’s discretion [13], with no established standards for ICE visualization in persistent atrial fibrillation ablation. Our study aims to determine feasibility and safety of LA ICE imaging guided ablation for persistent AF with the goal to establish procedural workflow allowing ablation strategy resulting in durable and limited number of lesions achieved due to superior LA ICE imaging.

Here, we report a case series of patients with persistent atrial fibrillation who underwent fluoroscopy-free catheter ablation with PFA guided by left atrial ICE at a single hospital center. We describe the detailed workflow of the procedure, as well as periprocedural and short-term outcomes.

## 2. Methods

### 2.1. Study Population

Thirty consecutive patients with persistent and long-standing AF undergoing pulse field ablation between October 2024 and January 2025 at our institution were included in the analysis. A total of 22 patients had no prior history of ablation, and repeated ablation was performed in 8 patients. There were no patients excluded from the study due severe comorbidities or other contraindications for AF ablation procedure under general anesthesia. The baseline patient characteristics are presented in Table 1. All patients provided written informed consent for the ablation procedure and data analysis, allowing their information to be used in this case series study. Our case series study was exempted by the Institutional Review Board due the retrospective nature of the analysis with de-identified data. The research reported in this paper adhered to Declaration of Helsinki guidelines on human studies.

### 2.2. Procedure Description and Workflow

All patients underwent pulse field ablation with a multielectrode Farawave catheter (Farapulse^TM^; Boston Scientific, Marlborough, MA, USA), with PVI and left atrial posterior wall (LAPW) isolation as the index procedure. In patients who remained in atrial fibrillation post-PVI and -LAPW isolation, cardioversion was performed, and then a high dose of isoproterenol was used to elicit non-PV triggers after confirming PV and LAPW isolation. Additional PFA was performed only in the presence of spontaneous or induced triggers or induced other atrial arrhythmias such as atrial flutter or tachycardia. Patients who underwent concomitant radiofrequency ablation in addition to PFA for right-sided flutter or supraventricular tachycardia were excluded from the analysis.

All procedures were performed under general anesthesia with uninterrupted anticoagulation. Intravenous heparin was administered to maintain an activated clotting time, with the goal of 350 sec during the procedure after obtaining IV access. Ensite Precision (Abbott, Abbott Park, IL, USA) was used for electroanatomic mapping, with the ViewMate™ Multi Ultrasound System and ViewFlex™ Xtra ICE Catheter (Abbott, Abbott Park, IL, USA) used for intracardiac imaging.

Our standardized approach to left-sided ICE-guided zero-fluoroscopy PFA workflow for persistent atrial fibrillation consists of the three steps listed below.

**Figure 1:**
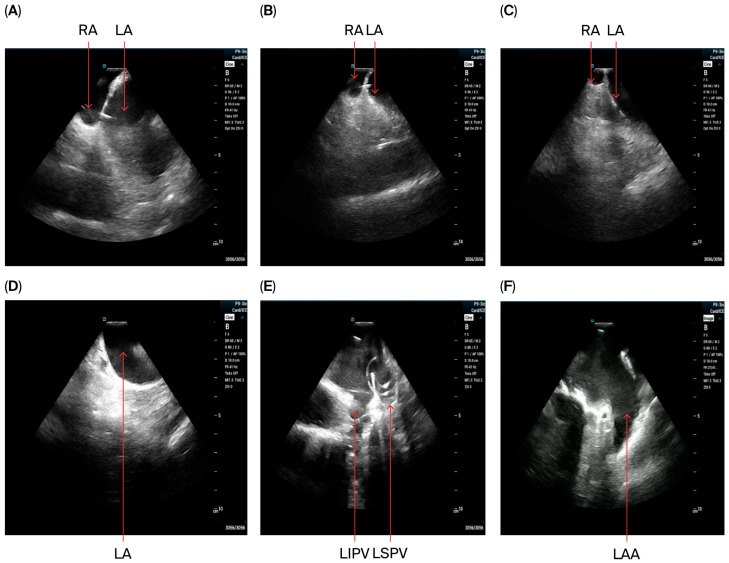
Placement of the ICE catheter in the LA. With a view of the interatrial septum in the RA, clockwise and counterclockwise rotations of ICE are performed to visualize the plane where the radiofrequency wire crossed the septum (**A**). Anterior tilt is applied to move the catheter towards the wire (**B**). Once the upper edge of the wire disappears from view (**C**), the ICE catheter is moved along the wire towards the LA and crosses the septum (**D**). Visualization of LA and left pulmonary veins with wire in LSPV (**E**) “Home view” for exclusion of LAA thrombus prior to ablation (**F**).

1. Catheter placement including transseptal catheterization and left atrial placement of the Faradrive sheath and ICE probe for direct LA visualization.

- Via the access in left femoral vein, the ICE catheter was advanced to the RA and the decapolar catheter was advanced to the coronary sinus. Baseline ICE images were obtained from the RA and then RV to assess the anatomy of the interatrial septum, LV function, and the presence of preprocedural pericardial effusion. Then, the ICE probe was advanced to the SVC to visualize the transseptal wire and sheath.
- Via the access in the right femoral vein, the VersaCross Access Solution wire (Boston Scientific, Marlborough, MA, USA) and sheath were advanced to the SVC under ICE monitoring. A single transseptal approach was performed and after crossing the septum VersaCross wire was placed in the left superior pulmonary vein, which was confirmed in ICE views from RA and from RV if needed.
- The VersaCross sheath was exchanged for the Faradrive sheath and dilatator. After predilatation of transseptal puncture, the Faradrive sheath was retracted, and the ICE catheter was advanced along the VersaCross wire to the left atrium (Figure 1A–D). Then, the Faradrive sheath was moved back to the LA, and the dilator and Versacross wire were removed, with the ICE catheter and PFA sheath placed in the LA via a single transseptal puncture.
- ICE images of the LA, including the LAA, were obtained from all patients to exclude LAA thrombus (Figure 1E).

2. Electroanatomic mapping and pulse field ablation with PVI and posterior wall isolation under EAM and ICE guidance.

- A circular mapping catheter (CMC) or high-density catheter (with repeated ablation) was advanced to the LA via the Faradrive sheath. Electroanatomic mapping of the LA, including detailed voltage assessment was performed. Then, over the J-tip wire, the Farawave pentaspline catheter was advanced to the LA via the Faradrive sheath. The 0.035-inch J-tip guidewire was attached to the EnSite mapping system’s pin box via a DuoMode extension cable (Boston Scientific, Marlborough, MA, USA) for additional visualization in LA and pulmonary veins. ICE catheter imaging was used to ensure appropriate catheter positioning and catheter–tissue contact with each PV and LAPW PFA lesion delivered. Prior to ablation, 0.2 mg of intravenous glycopyrrolate was administered to all patients to avoid vagal reactions during PVI.
- PV isolation was performed with the PFA catheter placed at the PV ostium, with 4 applications in the basket configuration and 4 in the flower configuration for a total of 8 applications per PV. Appropriate rotation of the catheter following 2 applications in each position was assured with LA ICE and EAM visualization. Two additional applications in the flower configuration were delivered at the operator’s discretion in the left and right carina, towards the LA ridge and septal aspect of the right pulmonary veins based on the anatomy and presence of atrial potentials.
- LAPW isolation was achieved with overlapping lesions placed in the flower configuration, with 2 application per site. With the J-tip wire in the left PV serving as a ‘hook’, rotating the catheter clockwise in the flower configuration allowed ablation of the posterior wall near the left veins; similarly, with the J-tip wire in the right pulmonary veins, rotating the catheter counterclockwise allowed appropriate contact for the posterior wall isolation near right veins. Remaining mid-posterior wall was ablated with overlying lesions in the flower configuration. ICE was used with each ablation application to ensure optimal contact.
- Cardioversion was performed if the patient remained in atrial fibrillation post-ablation. PV isolation was confirmed by the presence of entrance and exit blocks in all PVs, while posterior wall isolation was determined by the complete abolishment of posterior wall potentials from the LA roof to just below the lower regions of the inferior PVs.

3. Testing for non-PV triggers and additional PFA of induced triggers and atrial arrhythmias.

- A high-dose isoproterenol challenge at a rate of 20 mg/min was performed in all patients unless the dose was not tolerated. If the presence of spontaneous or induced non-PV triggers or atrial arrhythmias was detected, further PFA was performed.
- For superior vena cava (SVC) isolation, the ICE catheter was retracted to the top right of the atrium to guide the ablation procedure. Prior to and after ablating the SVC, the CMC was placed at the junction of the SVC and right atrium to map the phrenic nerve and to detect potential phrenic nerve injury. Then, the Pentaspline catheter was placed at the junction of right atrium and SVC (the lower edge of the right pulmonary artery level in ICE imaging). A total of 4 applications in the basket configuration were delivered (2 followed by 2 other applications after rotating the catheter 30 degree), and heart rate was monitored during the procedure.
- To ablate induced perimitral flutter, an anterior mitral line was created using PFA lesions delivered in the flower configuration, with 3 applications per site from the mitral annulus to the right superior pulmonary veins. Intravenous nitroglycerin was given prior to PFA, with dosing based on a previously published protocol [14]. The ST segment was monitored during and post-ablation.
- For ablation of induced focal atrial tachycardias, high-density catheter was used for activation mapping, and PFA was delivered in 3 applications in the flower configuration in each targeted site.

Upon completion of the procedure, ICE imaging was repeated to ensure the absence of new pericardial effusion. All patients were under continuous telemetry and monitored for complications during the 24 h stay in the hospital, and they were discharged with an antiarrhythmic drug, which was subsequently discontinued after a 2-month.

### 2.3. Follow-Up

Follow-up was performed at 2 weeks and at 3 months in the EP Clinic and within 6 months in the cardiology office, with Holter monitoring in all patients at 3 months. Patients were instructed to report any clinical symptoms of AF or any observations via wearable devices. A total of 24 out of the 30 patients monitored their heart rhythms with smartwatches with AF detection capabilities or the Kardia Mobile Device (AliveCor, Mountain View, CA, USA).

## 3. Results

**Figure 2:**
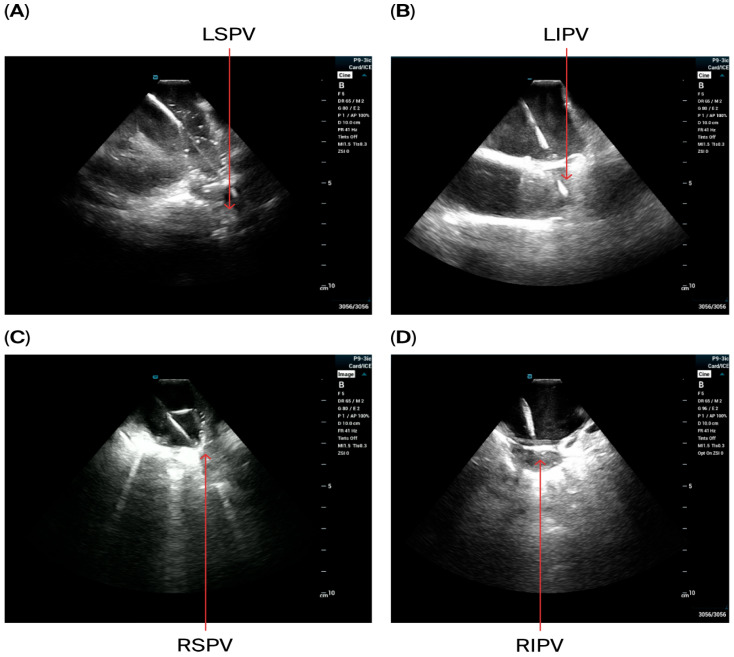
The position of the pentaspline PFA catheter in basket and flower configuration in LSPV (**A**), LIPV (**B**), RSPV (**C**), and RIPV (**D**), with catheter–tissue visualization with left-sided ICE.

**Figure 3:**
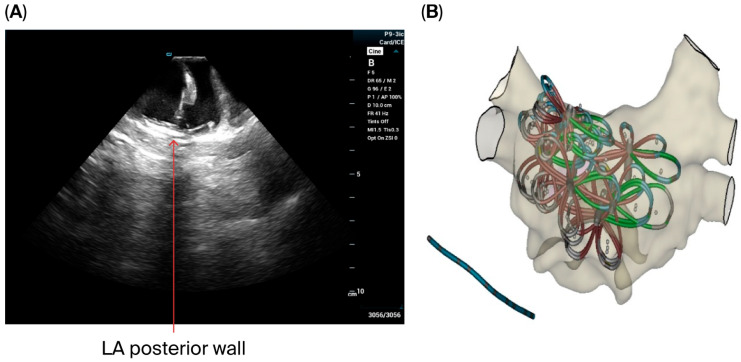
The position of the pentaspline PFA catheter during posterior wall isolation with catheter–tissue visualization with left-sided ICE (**A**) and correlating overlapping lesions in the EAM images (**B**).

All 30 patients in our series underwent procedures guided by EAM and ICE visualization from the LA. The ICE catheter was successfully placed in the LA in all cases, with satisfactory visualization of the LAA, PVs, and anterior and posterior LA structures. Additionally, appropriate pentaspline catheter contact during PVI, and posterior wall isolation was confirmed at each site targeted with pulse field ablation. ICE imaging from LA-guided AF ablation with a pentaspline pulse field ablation catheter is presented in Figure 2 and Figure 3.

**Figure 4:**
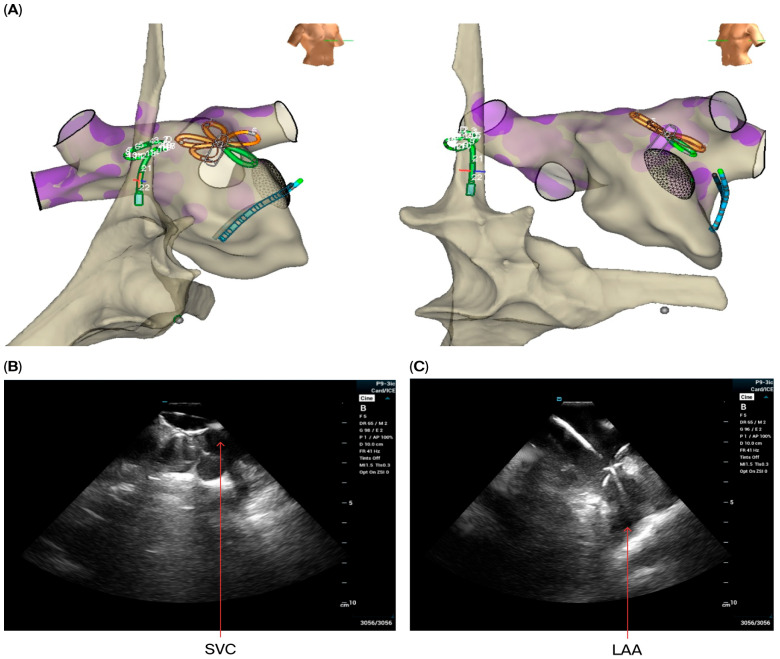
Visualization of CS, CMC and PFA catheters in EAM during testing for non-pulmonary triggers (**A**) and pentaspline catheter in ICE during ablation of non-pulmonary trigger from the SVC (**B**) and induced atrial tachycardia originating from the base of the LAA (**C**).

Isolation of the pulmonary veins and posterior wall was achieved using PFA guided by LA ICE and EAM in all 30 patients. However, four patients had additional spontaneous arrhythmias or extrapulmonary triggers which were induced post-ablation, including two patients with a perimitral flutter, one patient with induced AT from the LAA base, and one patient with an extrapulmonary trigger induced from the SVC treated using PFA, as described above. Examples of use of ICE imaging for PFA of extra-pulmonary triggers and ablation of induced atrial tachycardia are presented in Figure 4.

The average procedure time was 91 min. There were no major complications, including pericardial effusion, stroke, phrenic nerve injury, myocardial infarction, or coronary spasm, or vascular complications requiring intervention. Early AF recurrence occurred in 5 out of 30 patients, and AAD was discontinued in 28 out of 30 patients at 2 months. The absence of atrial fibrillation recurrence was confirmed with negative Holter monitoring in 27 out of 30 patients within 3 months post-ablation. Clinical follow-up at 6 months showed no recurrence in 25 out of 30 patients’ post-ablation. The procedural and post-procedural outcomes are presented in Table 2.

## 4. Discussion

Due to the importance of catheter–tissue contact visualization for effective PFA lesion delivery, there is growing interest in the use of ICE to guide imaging with this procedure. With PFA becoming a mainstream technology for the ablation of AF, there is increasing evidence of potential deleterious effects of nontargeted atrial tissue injury and hemolysis by electroporation [15], which is at least partially driven by non-optimal catheter–tissue contact. The goal of our study was to determine the efficacy and safety of using LA ICE imaging for superior catheter–tissue contact during PFA for persistent AF. Through our series, we present a standardized workflow, using a pentaspline ablation catheter for PFA of persistent atrial fibrillation with left-sided ICE and EAM. We performed pulmonary vein isolation and posterior wall ablation as an index procedure, followed by additional ablation if arrhythmia or other extrapulmonary vein triggers were induced. We established the above workflow after performing previously zero-fluoroscopy thermal ablation procedures with ICE and EAM guidance and conducting two PFA procedures with fluoroscopy and right-sided ICE. Despite limited prior experience, our procedural times were not significantly different than those reported in other studies [13,16] and had decreased over time. Procedural efficacy was confirmed with the complete isolation of the pulmonary veins and posterior wall, determined using a dedicated mapping catheter at the end of the procedure. The short-term success rate, with no documented recurrence at 3 months and no clinical recurrence at 6 months, was comparable to the results of large-scale studies using pulse field ablation for persistent atrial fibrillation. As an example, in the ADVANTAGE AF, a large prospective study of PFA for persistent atrial fibrillation using a strategy of PVI and LAPW isolation with fluoroscopy, the rate of freedom from symptomatic atrial fibrillation was 85.3% at 1 year [13]. Furthermore, there were no acute periprocedural complications in our series supporting the safety of the left-sided ICE workflow. No increase in risk of complications during AF ablation procedure performed with LA ICE imaging as compared to routine RA ICE imaging had been previously described, including no increase in thromboembolic events or risk of pericardial effusion [9].However, risk of thromboembolism and air embolism associated with additional transseptal sheath and catheter placement in LA as well as cautious maneuvering of the ICE catheter in LA should be always considered while performing procedures with LA ICE imaging. Additionally, retracting ICE catheter in LA closer to transseptal site at the time of electrical cardioversion is a routine in our practice.

Although a study focusing on PFA PVI in paroxysmal atrial fibrillation showed no improvement in procedural metrics with the addition of ICE to fluoroscopy [17], a recently published prospective analysis on ICE-guided PFA for paroxysmal and persistent AF with confirmed catheter–tissue contact showed a better success rate and a lower rate of reconnections [13]. The differences in the above studies likely result from the crucial distinction between using ICE to support the procedure versus guiding the catheter with the assurance of appropriate catheter–tissue contact during ablation. Visualizing LA structures from the RA is usually adequate as reported in the aforementioned studies; however, significant variability in the ICE imaging quality of left atrial structures exists, especially of the right superior pulmonary vein and posterior wall. In our experience, detailed visualization of ablation catheter–tissue contact is superior with left atrial ICE-guided PVI compared to that with right-sided ICE; however, the most significant benefit of left-sided ICE is observed during posterior wall ablation and sites outside pulmonary veins. Confirmation of an appropriate contact, with a detailed visualization of each lesion on the posterior wall, led to a significant decrease in the number of overlapping ablation lesions during procedures over time and was overall lower in our study than reported in other studies using right-sided ICE or only optionally left-sided ICE imaging [13]. LA ICE imaging resulting in limitation of electroporation during the PFA may potentially decrease the risk of unnecessary nontargeted tissue injury and hemodialysis during PFA procedures, which carries unknown long-term consequences. With PFA of AF guided by ICE imaging proved to be effective and safe strategy for ablation of nonpulmonary triggers such as superior vena cava [18], which we incorporated in our workflow and with further improvement of LA ICE imaging technique during PFA of AF with successful use of 4-dimensional LA ICE described [19], non-fluoroscopy PFA of AF has become vital alternative to PFA of AF using traditional imaging.

PFA PVI with a pentaspline catheter could be performed with ICE imaging as a fluoroscopy-free procedure; however, the addition of EAM serves as an additional tool to ensure appropriate catheter positioning and rotation as well as improve the safety of the zero-fluoroscopy procedure. Additionally, EAM use is crucial in persistent AF procedures including the treatment of nonpulmonary triggers and other atrial arrhythmias induced post PVI and LPWA. As we showed in our study, using nonproprietary EAM systems with a pentaspline catheter system is feasible, thus broadening the choice of procedural tools that can be used with this pentaspline PFA catheter. Avoiding radiation exposure by excluding the LAA thrombus with left-sided ICE rather than preprocedural CT imaging and not using fluoroscopy during the procedure are additional benefits of the above procedural approach.

### Study Limitations

This study analyzed retrospectively outcomes of LA ICE imaging guided PFA for persistent atrial fibrillation with no direct comparison with other imaging modalities. With only the pentaspline PFA catheter used, our study does not provide insight into the outcomes with other PFA technologies for ablation of persistent AF. The small sample size, collection of data from procedures performed by single operator and in one hospital center represents additional limitations of this study.

## 5. Conclusions

Fluoroscopy-free ablation guided by left-sided ICE for persistent atrial fibrillation can be performed with superior catheter–tissue contact imaging in a safe manner and with a comparable procedural time and short-term efficacy as reported with the use of other imaging modalities.

## Figures and Tables

**Table 1:** Baseline patient characteristics.

Group size	30 patients
Sex distribution	12 females 18 males
Mean age (range)	70.3 (41–81 years old)
Average time since diagnosis of atrial fibrillation (range)	3.5 years (3 months–12 years)
Prior use of antiarrhythmics (%)	23 patients (77%)
Patients who had prior AF ablation (%)	8 patients (26%)

**Table 2:** Procedural and post-procedural outcomes.

Number of procedures completed (%)	30 (100%)
Successful isolation of the PVI + posterior wall (%)	30 (100%)
Other ablation sites (%)	4 patients (13%) - SVC isolation (1 patient) - LAA AT (1 patient) - Anterior mitral line (2 patients)
Average procedural time (skin-to-skin)	91 min (57–147 min)
Average number of PFA applications - First ablation - Repeated procedure	54 applications 58 applications 42 applications
Major complications	None
30-day readmissions (%)	1 patient (3.3%)
Early AF recurrences within 30 days	5 patients (16.6%)
AAD discontinued at 2 months	28 patients (93.3%)
Free from AF at 3 months (%)	27 patients (90%)
Absence of clinical recurrence at 6 months (%)	25 patients (83.3%)

## Data Availability

The original contributions presented in this study are included in the article. Further inquiries can be directed to the corresponding author.

## Abbreviations

The following abbreviations are used in this manuscript:

PFA	pulse field ablation
AF	atrial fibrillation
ICE	intracardiac echo
EAM	electroanatomic mapping
PVI	pulmonary vein isolation
LAPW	left atrial posterior wall ablation
RA	right atrium
LA	left atrium
